# Bayesian Network Analysis reveals resilience of the jellyfish *Aurelia aurita* to an Irish Sea regime shift

**DOI:** 10.1038/s41598-021-82825-w

**Published:** 2021-02-12

**Authors:** Emily G. Mitchell, Margaret I. Wallace, V. Anne Smith, Amanda A. Wiesenthal, Andrew S. Brierley

**Affiliations:** 1 Pelagic Ecology Research Group, Scottish Oceans Institute, Gatty Marine Laboratory, School of Biology, University of St. Andrews, St Andrews, KY16 8LB Scotland UK; 2 Centre for Biological Diversity, Sir Harold Mitchell Building, School of Biology, University of St. Andrews, St Andrews, KY16 9TF Scotland UK; 3 Present Address: Department of Zoology, University of Cambridge, Cambridge, CB2 3EJ UK; 4 Present Address: Scottish Qualifications Authority, Optima Building, 58 Robertson St, Glasgow, G2 8DQ UK; 5 Present Address: Pharmaceutical Biology, Saarland University, 66123 Saarbrücken, Germany

**Keywords:** Community ecology, Conservation biology, Ecological networks, Ecosystem ecology

## Abstract

Robust time-series of direct observations of jellyfish abundance are not available for many ecosystems, leaving it difficult to determine changes in jellyfish abundance, the possible causes (e.g. climate change) or the consequences (e.g. trophic cascades). We sought an indirect ecological route to reconstruct jellyfish abundance in the Irish Sea: since zooplankton are jellyfish prey, historic variability in zooplankton communities may provide proxies for jellyfish abundance. We determined the Bayesian ecological network of jellyfish–zooplankton dependencies using jellyfish- and zooplankton-abundance data obtained using nets during a 2-week cruise to the Irish Sea in 2008. This network revealed that *Aurelia aurita* abundance was dependent on zooplankton groups Warm Temperate and Temperate Oceanic as defined by previous zooplankton ecology work. We then determined historic zooplankton networks across the Irish Sea from abundance data from Continuous Plankton Recorder surveys conducted between 1970 and 2000. Transposing the 2008 spatial dependencies onto the historic networks revealed that *Aurelia* abundance was more strongly dependent over time on sea surface temperature than on the zooplankton community. The generalist predatory abilities of *Aurelia* may have insulated this jellyfish over the 1985 regime shift when zooplankton composition in the Irish Sea changed abruptly, and also help explain its globally widespread distribution.

## Introduction

Over the last few decades it has become increasing apparent that jellyfish play crucial roles in marine ecosystems^1–4^. Whilst jellyfish used to be considered merely a trophic dead-end with few predators, through the use of new approaches such as stable isotope analysis, metabarcoding and ‘critter cams’ we now know that jellyfish are embedded in complex networks of trophic interactions^5^. However, understanding how these jellyfish interactions change through time is hampered because jellyfish abundance data are sparse compared to fish stock assessments, fisheries landing and mesozooplankton records. With a few notable exceptions^2^ there is a general lack of long-term (> 60 years) data on jellyfish abundance^2,6^. This paucity of knowledge is partly because jellyfish lack hard structures (scales, teeth, bones) that can leave some lasting record of historic abundance, partly because of sampling difficulties (the fragile bodies of jellyfish are broken by nets), and partly because jellyfish have been dismissed as a nuisance or worse by most researchers conducting the regular and geographically wide-reaching surveys of abundant economically valuable fish stocks. The scarcity of historical data limits the predictions that can be made of drivers of changing jellyfish abundance, potential tipping points in jellyfish ecosystems, or of consequences to fisheries of variation jellyfish abundance^2,6,7^.

It has been argued that jellyfish ecology should be incorporated in to ecosystem-based approaches fishery management because jellyfish can compete directly in each of their life cycle stages with some fish for zooplankton^8–12^, and because jellyfish can predate fish eggs and larvae^13^. Of major concern is the potential for the so-called ‘rise of slime’ whereby jellyfish come to dominate ecosystems as a consequence of reduction of fish stocks by fishing and by marine habitat degradation^14^. It is now becoming increasingly accepted that jellyfish ‘blooms’ can have substantial negative consequences for fish and fishing^1^. Fisheries face major financial losses when jellyfish blooms occur in their fishing grounds: these losses can be due to equipment damage, decreased quality and quantity of catch, or even a complete failure of harvest^3,11,15^. In one of the few situations where jellyfish abundance data have been collected during fishery surveys^5^, significant negative links between fish recruitment and jellyfish abundance have been exposed^4^.

Effects of jellyfish on fish stocks may be direct or indirect, and/or be impacted by wider ecosystem changes. These ecosystem changes may be gradual, such as the creeping changes in the distribution of species in the face of warming, or more rapid as ‘regime shifts’ that see wholesale change in community compositions^1,2,4^. Regime shifts in the phyto- or zooplankton communities such as in the northern Benguela upwelling system have been shown to potentially impact jellyfish abundance and fish stocks^9,16^. Therefore, it is key for fisheries management and marine ecosystem-based management more broadly, and indeed for quantification of fundamental biogeochemical processes such as carbon cycling, to understand the timing, causes and consequences of changing jellyfish abundance^11,17^. For example, in the Benguela Current Large Marine Ecosystem, overfishing caused a regime shift from a fish-dominated system to a jellyfish-dominated system^3,16^. Whether this shift is reversible remains to be seen but, for fundamental ecological understanding and for management of a sometimes commercially important marine living resources, it is important to understand how jellyfish populations change through time, and to understand if these are in concert with or independent from changing temperatures or changing zooplankton communities^1,18–22^.

**Figure 1:**
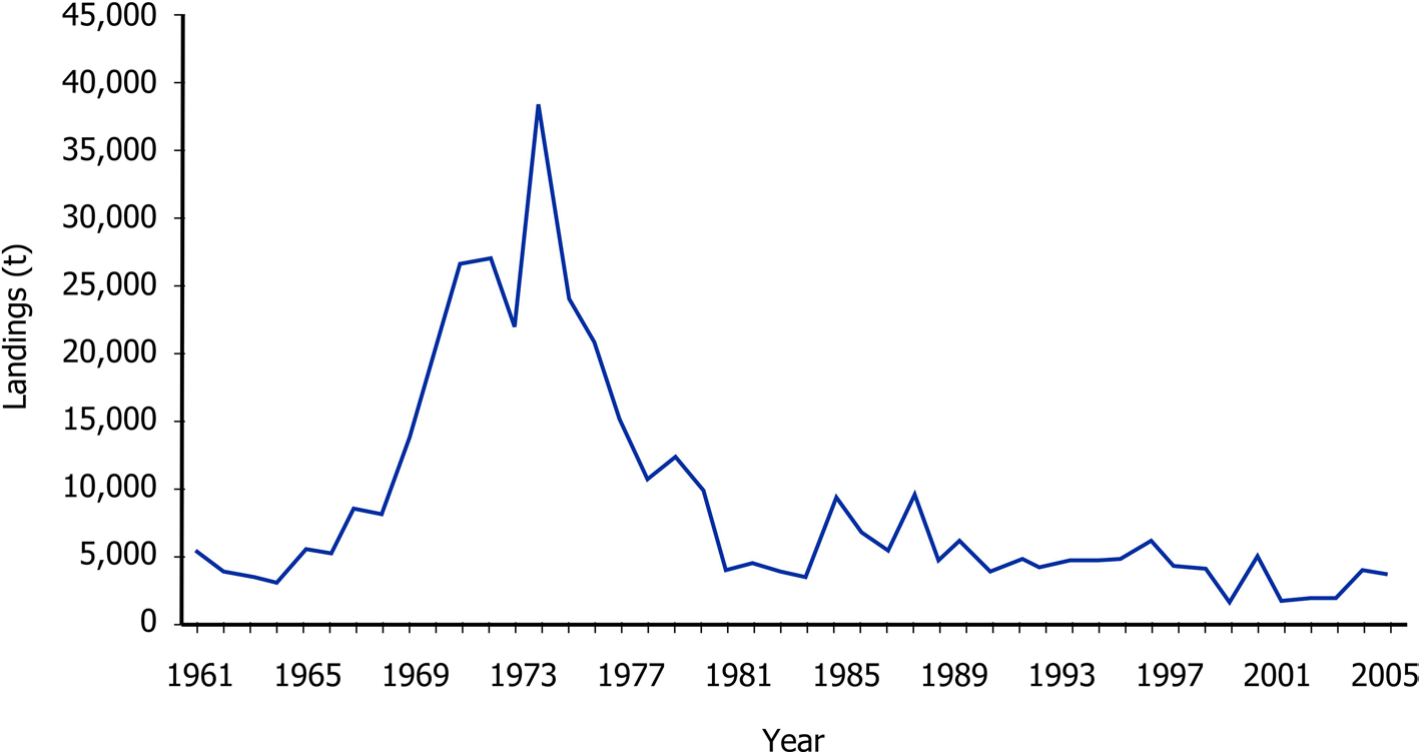
Herring landings data for the Irish Sea (modified from Ref.^27^). X-axis is time measured in years and y-axis are landings in metric tonnes.

In this study we develop a new approach to infer timeseries of jellyfish abundance from historical zooplankton data, data that were in this instance gathered by the Continuous Plankton Recorder (CPR)^23,24^. We focussed on the Irish Sea because this region has historically been a productive and commercially important fishing ground^25^ although herring stocks there crashed in the late 1970s (Fig. 1) and have declined steadily since, reaching an all-time low in 2019^26,27^. Between 1970 and 2000 zooplankton abundances have also changed dramatically: a regime shift around 1985 saw a 10° biogeographic shift of the mesozooplankton community towards the north^28,29^, and rapid declines in gadoid and salmon catches^30^. The Irish Sea has been something of a focus for jellyfish research^1,31–33^, but relationships between jellyfish, zooplankton and herring abundances remain largely unresolved because of the lack of a jellyfish timeseries: we set out to determine such a timeseries.

The logic underpinning our approach was that if jellyfish in the Irish Sea had a significant association in space with particular zooplankton (species or suites of species) that we could determine by paired jellyfish and zooplankton field sampling, then it may be possible to infer historic jellyfish abundance from historic zooplankton data. Such an historical approach would be potentially widely applicable because there are rich historic zooplankton records for many regions globally.

One method for understanding ecosystem dynamics is to consider the whole ecosystem as a network where different species or groups of species are defined as “nodes” and where correlations between them are described as “edges” which link correlating species together^34,35^. Correlations between species can be purely trophic, in which case the network represents a food web^36^, or can include other sorts of ecological interactions such as facilitation or competition for resources^37^. Including physical variables such as temperature or—in the aquatic realm—depth, also enables mutual habitat associations to be found^38^. Such multi-process networks can be reconstructed statistically using methods such as Discrete Bayesian Network Inference Algorithms (BNIAs) which can find network structures, including non-linear dependencies between nodes. BNIAs have primarily been used to calculate gene regulatory and neural information-flow networks^39,40^, but more recently have been applied successfully to reconstruct ecological^35,41^, palaeontological^42^ and—in the marine realm—to abyssal plain^43^, Antarctic^38^ and deep-sea benthic networks^44^. To the best of our knowledge this study is the first application of BNIAs to a pelagic ecosystem. It is important to note that the networks found by the BNIAs reflect dependencies caused by co-localisations (e.g. two species have high abundances) and not by any particular biological interaction, for example predation. The use of BNIA with ecological datasets enables direct dependencies (i.e. causal relationships) between nodes (here groups of species) to be found, with autocorrelation (i.e. mutual indirect correlations) between the nodes minimised. For example, if there are direct dependencies between species A and species B, and between B and C, it is likely that there is also an indirect dependency between A and C: the BNIA would only report the direct dependencies (as two edges), not the indirect one. BNIAs can detect direct positive correlations, which for spatial data indicate spatial co-occurrence, that are quantif by a positive Influence Score (IS). Negative correlations have a IS < 0 and represent a negative correlation or spatial segregation such that when the abundance of one species is high, the abundance of the other species will be low. Associations between the two species will be either positive or negative, dependent on the species abundances are coded within BNs as IS = 0. Thus, BNs presents information on both magnitude and direction of interactions between variables and select only those interactions that are direct. These generated BNs can be used to infer how the network is likely to change under different scenarios.

In this study, we first determine jellyfish–zooplankton interacts by using BNIAs to infer contemporary (2008) spatial jellyfish–zooplankton networks in Irish Sea field data. Secondly, we built zooplankton networks (using weekly data grouped into three decades 1971–2000) using historic Irish Sea data from the Continuous Plankton Recorder, and then thirdly we inferred historic jellyfish abundances (1971–2000) by applying the contemporary jellyfish–zooplankton interactions to the historic zooplankton networks. It was our hope that these analyses would enable us to infer how jellyfish abundances may have changed historically in the Irish Sea over a period when a regime shift occurred and the herring fishery collapsed^27,45^.

## Methods and materials

### Contemporary (2008) Irish Sea spatial jellyfish and zooplankton data

**Figure 2:**
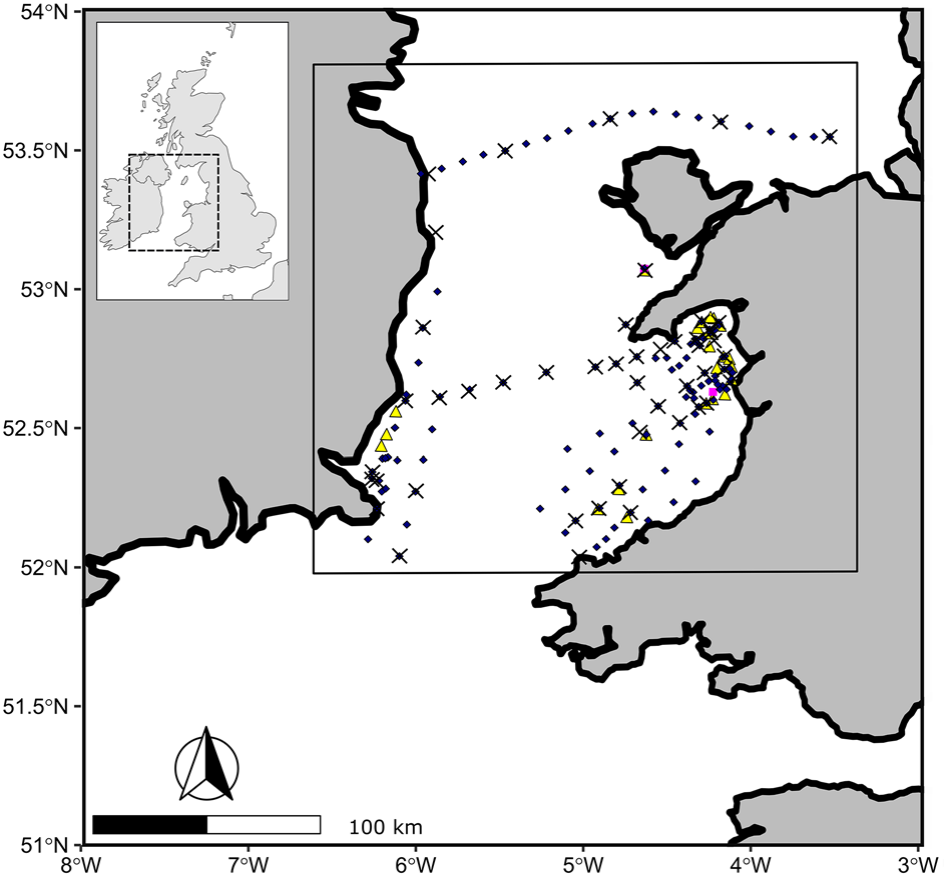
Cruise track in May 2008 showing locations for MIK net (pink squares), Otter trawl (yellow triangles), ring net (dark blue diamonds) and CTD casts (red crosses).

The data for the contemporary jellyfish–zooplankton network were collected over a 2-week cruise in the Irish Sea on the RV *Prince Madog* in May 2008 (Fig. 2).

#### Net sampling

To determine the mesozooplankton community composition, and abundance of jellyfish (pelagic medusae of the Cnidarian Classes Hydrozoa and Scyphozoa—these are technically zooplankton, but we call them jellyfish here for the sake of clarity), net sampling was undertaken using a horizontally-towed midwater MIK net, a vertically hauled 1 m diameter ring net, and an otter trawl (jellyfish only). A total of 144 stations were sampled (Fig. 2). Zooplankton were identified (we encountered a total of 111 species) and counts per sample were standardised to counts per unit volume with reference to flowmeter data that enabled volumes of water filtered during each haul to be determined. Two MIK net samples contained *Aurelia aurita* and *Cyanea capillata*, as did 65 ring net samples (see supplementary data). Of the 23 otter trawl deployments, (see Fig. 2) 18 contained *Aurelia,* 8 contained *Cyanea lamarckii*, 4 contained *Cyanea* *capillata* and 2 contained *Rhizostoma pulmo*.

#### Visual, acoustic and aerial sampling for jellyfish

**Figure 3:**
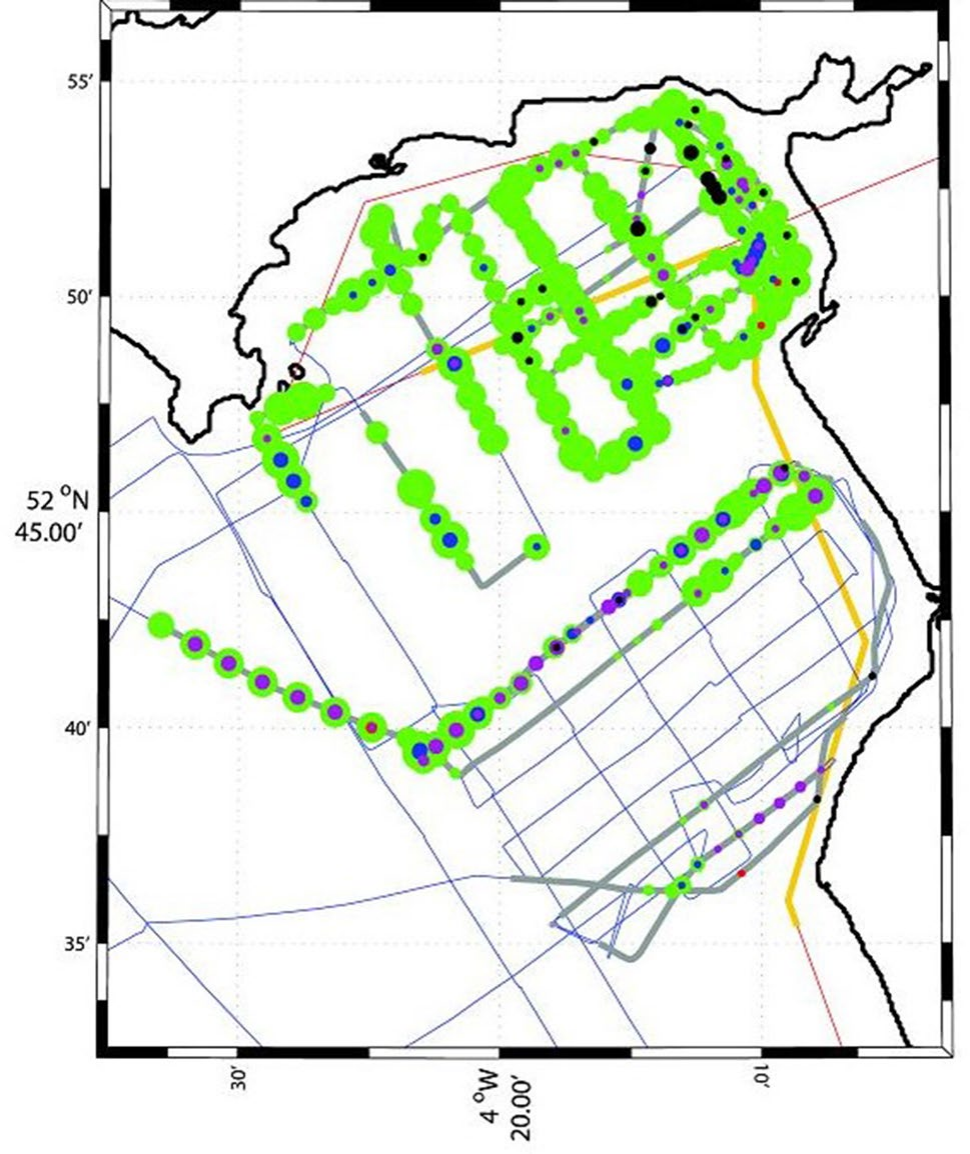
Jellyfish abundance sampling along the cruise route. Coloured circles are different jellyfish species: green *Aurelia;* red *Chrysaora hysoscella*; blue *Cyanea lamarckii*; purple *Cyanea* *capillata* and black *Rhizostoma pulmo*. The size of the circles is proportional to the log jellyfish count, varying between 0 and 9. The cruise track is given by the blue lines; observations the green line; the red line shows the aerial survey flight path, and the yellow line is a zone identified by previous aerial surveys as a jellyfish ‘hotspot’.

In addition to net sampling, efforts were made to obtain abundance data for jellyfish by visual counts from the foredeck cf.^4^, scientific echosounding (18, 38 and 120 kHz; Fig. 3 cf.^46^) and aerial survey^32,47^. Visual observations of jellyfish were made from the bow during daytime only. To derive categorical estimates of abundance for each station the visual observations that occurred 750 m before the station and 750 m after the beginning of each station were extracted, then the counts per distance were placed into Low, Medium and High categories.

Visual observation data were available in daylight only, and were heavily influenced by sea state and glare, so were not used in network analysis. Acoustic data were also ultimately discarded because the combination of transducer depth (transducers were in the vessel hull) and acoustic near-field effects^48^ left the upper c. 10 m unsampled. The aerial survey coincided with a day of very high wind, and sea surface conditions turned out not to be conducive to quantitative sampling: counting jellyfish is a tricky business!

### Historic zooplankton data

**Figure 4:**
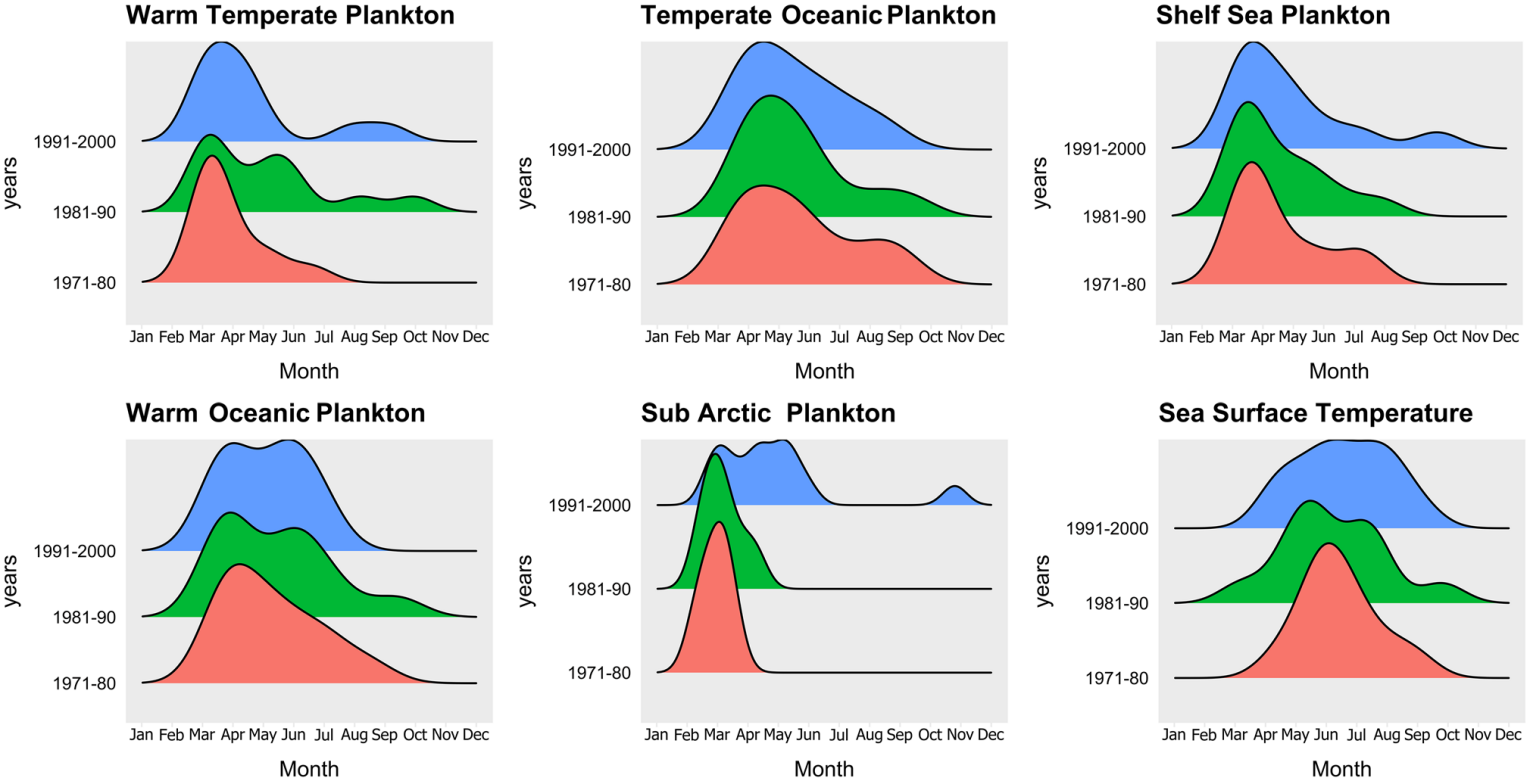
Historic Plankton group abundances (from CPR data) and sea surface temperature (from UK Meteorological Office). The three time periods are shown in different colours: Peach 1971–1980; Green 1981–1990, and Blue 1991–2000. The x-axis is the month of the year, and the y-axis shows the relative zooplankton abundance (scaled relative to each groups maximum) for each time period. This relative scaling is to enable clear comparisons of relative abundance between groups for each biogeographical zooplankton group over the three decades.

Historic zooplankton data were obtained from the Sir Alistair Hardy Foundation for Ocean Science (SAHFOS) Continuous Plankton Recorder (CPR) survey, and consisted of tow data obtained between 1971 and 2000 on the Dublin—Liverpool route (50°40′–56°N, 15–2°30′W). The CPR is towed at a depth of approximately 7 m but may actually sample the top 20 m due to turbulence^49^. Further details of CPR methods are given in Refs.^23,50,51^. Each CPR sample records the number of zooplankton present in approximately 3 m^3^ of water. 537 species of zooplankton are routinely identified by the analysts, and counts are standardised to take account of differing volumes of water filtered per sample^52^. For this study we considered abundant 24 morpho-groups routinely detected by the CPR in the Irish Sea. They consisted of: 9 species, 7 larvae groups, Isopods, Chaetognatha, Copepod nauplli, *Calanus* i-iv, *Pseudocalanus* adults, *Para-pseudocalanus* spp. and PCI (Chlorophyl) as a proxy for phytoplankton concentration (see supplementary data). For simplicity these groups (apart from PCI) will heron be referred to as zooplankton. We collapsed them into the five biogeographic groups identified previously^28,53^: Warm Temperate, Warm Temperate Oceanic, Temperate Oceanic, Shelf Sea and Sub Arctic (Fig. 4).

#### Data considerations for Bayesian Network Inference Algorithms

The Bayesian Network Inference Algorithm (BNIA) ingests discrete data to ensure that noise is masked and only the relative abundances of each taxon are important^34,35,39,40,54^. Calculation of the best Bayesian network given a data set is computationally intractable, so BNIA uses a search technique which explores different networks and returns the highest-scoring network encountered as the solution^55^. Zooplankton with no strong biogeographical affinities were removed from the analysis, namely Copepod nauplii, *Calanus* i–iv, Isopoda, Larvacea, Lamellibranchia, Echinoderm and Polychaete larvae, Chaetognath and *Limacina retroversa.* We combined the remaining 14 zooplankton species into five groups as described below (Table 1)^54^. In order to avoid Type I errors, often associated with high-levels of zero count data, we performed contingency-test filtering. This filtering disallowed an edge between two variables whose joint distribution showed no evidence of deviation from the distribution expected from their combined marginal distributions (chi-squared tests, *p* > 0.25)^35,39,40,54^ i.e. those which were correlated due to high levels of mutual zeros.

**Table 1:** Biogeographical zooplankton groupings following Ref.^53^ used in this study.

Biogeographical group	Species present in group
Warm Temperate	*Cyphonautes* larvae
Warm Temperate Oceanic	*Calanus helgolandicus*, Decapod larvae, Cirripede larvae, Euphausiids
Temperate Oceanic	*Acartia* spp., *Podon* spp., *Evadne* spp.
Shelf Sea	*Pseudocalanus, Para-Pseudocalanus* spp., *Temora longicornis,* fish larvae
Sub Arctic	*Calanus finmarchicus, Tomopteris* spp.

The data were grouped as follows:

Step 1: Zooplankton Grouping. The zooplankton species were grouped by their biogeographical association (cf.^28,29,45^; see Table 4) to enable us to determine the effect (if any) of the North Atlantic regime shift on the networks.
Step 2: Weekly Averaging. Weekly averages were taken of the CPR count data to ensure consistent networks. The number of CPR samples varied between zero and fifteen per week.
Step 3: Temporal Grouping. Previous research indicates that the regime shift occurred in the mid-1980s, with the exact year of varying between species examined and analysis type^28^. We split the data into three temporal groups, 1971–1980, 1981–1990 and 1991–2000 in order to ensure that the entire potential regime shift window was contained within one temporal group (Fig. 4).

#### Incorporation of environmental data in BNIA

We included sea-surface temperature (SST) and an index of the North Atlantic Oscillation (NAOI) as environmental variables in the zooplankton networks. SST data were obtained as monthly medians from the UK Meteorological Office^56^, and weekly values were calculated by interpolation from those. The NAOI was defined as the difference between the normalised sea level pressure over Gibraltar and the normalised sea level pressure over the southwest UK, and monthly indexes were obtained from The Climate Research Group, University of East Anglia (http://www.cru.uea.ac.uk/ftpdata/nao.dat). The data we used were as calculated by Ref.^57^.

### Bayesian network inference

Bayesian network inference (BNI) was performed in Banjo^41^. Data preparation for Banjo (grouping and discretisation) and statistical analyses were performed in R v 3.3.2 cf.^58^. Further analysis of Banjo outputs, when required, used the functional language Haskell^59^. The scripts are available on Github: https://github.com/egmitchell/bootstrap. R code is available a thttps://github.com/egmitchell/jellyfish.

To determine each Bayesian network, the four steps below were followed as per Ref.^35^:

1. Discretisation. We split the data into three intervals; zero counts, low counts and high counts. Low counts consisted of counts below the median for the species group and high counts were counts over the median. Medians were used rather than means because for some groups the high counts were very high and use of the mean would have resulted in a very small number of samples grouped in the highest interval. Employing a large number of bins preserves resolution in the dataset, while fewer bins provide more statistical power, and greater noise masking: three bins has been found to be optimal for ecological data^35,39,54^. Zeros were treated as a separate entity because the absence of individuals (i.e. zero presence) is ecologically very different to the presence of just one: this difference is in contrast to zero versus one in measures of gene expression, which varies on a continuous scale between zero and one^54^ (Table 2).
2. Contingency-test filtering. The CPR dataset (6010 trawls obtained from between 1 and 4 times per week, with 23 zooplankton and PCI (Chlorophyll Concentration) contained a large number of zeros (56%), which could potentially have created problems with false positives (Type I errors) due to autocorrelation between these zeros. We combated this problem in three ways. 1) By grouping zooplankton into biogeographic groups, instead of just using the individual species data. 2) By taking weekly averages and having zero counts as a separate bin we reduced the number of zeros to 39%: the BNIA we used requires that the data are evenly distributed across all the bins, so 39% is adequate for a 3 bin system. 3) We used Chi-squared filtering to remove edge pairs which showed no evidence of deviation from the distribution expected from their combined marginal distributions (chi-squared tests, *p* > 0.25 cf.^35^).
3. BNIA. Banjo was run on each data set to search 10 million possible networks using a greedy search, whereby at each search point the optimal edge addition to the network was used. The number of possible parents for each node (the edges that feed into a node) was limited to three to help eliminate artefacts^54^. For each edge the influence score (IS) was calculated.
4. Model Averaging. If a network suggested by Banjo represented the underlying ecological network, we would expect the same network to persist even if a few sample points (under 1%) were removed from the input data. Note that this bootstrapping removes the weekly samples, rather than individual species or groups. We found, however, that there was variation within the suggested networks when a small number of sample points were removed, so we applied a model averaging to the networks to overcome this stochasticity. For each time period, we took 100 random samples, consisting of 90% of the total available data points. For each edge that occurred, the probability of occurrence was calculated as the number of times the edge appeared over the total number of 100-random-sample bootstraps conducted. This probability distribution of node frequencies was bimodal for each of the network (2008, 1970s, 1980s, 1990s). These bimodal distributions suggest that there are two Gaussian distributions of edges, the rare/low occurrence ones, and the highly probable edges. The mean IS for each edge over all bootstraps was also calculated.

**Table 2:** Zooplankton count data for the different variables included in the Bayesian network inference.

	Warm Temperate plankton	Warm Temperate Oceanic plankton	Cold Temperate plankton	Shelf Sea plankton	Sub Arctic plankton	Chlorophyll Concentration	NAO	Sea Surface Temperature	Herring abundance
**70s**									
Median	0.71	15.59	72.08	148.83	0.25	0.84	0.13	11.74	21.14
Zero	2	0	0	0	0	0	0	0	0
Low	4	5	5	5	5	5	6	5	5
High	4	5	5	5	5	5	4	5	5
**80s**									
Median	2.38	12.71	31.73	114.66	0.33	0.81	0.27	10.15	4.92
Zero	4	0	0	0	0	0	0	0	0
Low	3	5	5	5	5	5	6	5	5
High	3	5	5	5	5	5	4	5	5
**90s**									
Median	1.69	10.03	31.16	72.39	0.40	1.01	0.19	9.95	4.56
Zero	4	0	0	0	0	0	0	0	0
Low	3	5	5	5	5	5	7	5	6
High	3	5	5	5	5	5	3	5	4

	*Aurelia aurita*	Other jellyfish	Chlorophyll Concentration	Cold temperate plankton	Sub artic plankton	Shelf sea plankton	Cold temperate oceanic plankton	Warm temperate plankton	Warm temperate oceanic plankton
**2008**									
Median	4	2	1005	3090	3	8073	8073	23	480
Zero	92	75	0	85	82	0	14	4	4
Low	12	21	61	19	19	61	46	53	56
High	15	19	61	19	15	61	60	55	57

Median is the median count numbers for each variable which defines the break between the High and Low category.

For the BNI analyses, jellyfish count data from net samples were split into two groups: *Aurelia aurita*, which was the most abundant species (97.8% by number), and ‘other taxa’ which consisted of *Cyanea* spp. (1.9%) and *Cosmetira pilosella* (0.3%). *Rhizostoma* were also caught, but due to very low numbers they were not included in the analyses. The zooplankton were grouped by biogeographic affinity cf.^28^ (as per Table 1) to ensure consistent networks. This biogeographic grouping was required because not all zooplankton species were present in all of the 144 samples, and zero-inflated data are not valid with the Bayesian priors used in this BNIA.

### Inference of historical jellyfish abundance

One of the most powerful aspects of BNs is the ability to make inferences of how the probability of one node (taxa or physical variable) being in each state (zero, low or high for a taxon) is likely to change given that another node is in a given state (zero, low or high for a taxon)^38^. This inference is made by calculating the probability of node A being in a given state given node B is in a given state. For example, the probability of a given species being in a zero, low or high abundance state can be inferred for SST being in either a low or high state. This inference can be used to calculate the likely consequence for species abundance of changing variables such as SST.

All nodes that have dependencies between A and B are included in the calculation:

$${\text{P}}\left( {{\text{A}}\left| {\text{B}} \right.} \right) = \sum\limits_{n = 1}^{n = N - 1} {\frac{{\prod\nolimits_{s = 1}^{s = S} {{\text{P}}\left( {{{\text{B}}_n}\left| {{A_{n + 1}}} \right.} \right)} }}{{\sum\nolimits_{m = 1}^N {P\left( {B\left| {{A_m}} \right.} \right)P\left( {{A_m}} \right)} }}}$$

The N are the total number of nodes in the network and *n* and *m* are the indices for the chain of nodes of length N connecting the first and last nodes. S are the number of discrete states for each node, which are indexed *s.* In order to infer the likely change of one taxon’s (A) abundance on another (B), for example if A changes from a high abundance state to a zero abundance state, the probabilities of B existing in all states are calculated given a high abundance state for A, and the probabilities for B existing in all states are calculated given a zero abundance state for A. The inferred change in one taxon’s abundance is then the difference between these probabilities.

The abundance probabilities for the biogeographical zooplankton groups (Warm and Cold Temperate) were calculated (e.g. *P(High abundanceǀLow Temperature)*) given the SST state for each of the three decades. The mean temperature in the Low SST state was approximately 4 °C lower than the High SST state. An increase in the probability of a species/group given the probability of another species/group having a zero count represents the increase or decrease in abundance in that area, for that temperature change.

In order to calculate historic jellyfish abundance probabilities, the dependencies of jellyfish on zooplankton were assumed to remain static over the three decades. Note the implicit assumption here that *Aurelia* continue to feed on the same biogeographic zooplankton groups, although the relative rate of feeding may change. For each decade the abundance probabilities of the biogeographic zooplankton groups that the jellyfish nodes depended on were calculated. The jellyfish node probability abundances (zero, low, high) given either Low SST or High SST were calculated for each decade given the dependencies found in the spatial network inferred from the 2008 field data.

### Using Naïve Bayes Classifiers to infer the relative importance of different zooplankton groups to herring

In order to determine the relationships between zooplankton groups and herring, we used naïve Bayes Classifiers to determine the relative importance of each zooplankton biogeographic group and environmental group to herring. The herring data were recorded as annual catch data for the period 1970–2000^27^, which meant there were only 10 samples for each decade: this was insufficient for direct incorporation directly into the CPR BNs, but naïve Bayes Classifiers can determine and rank the importance of each group relative to each other^60^. Herring have a multi-year life-cycle, and there are lags between spawning and recruitment and capture. Impact by jellyfish on herring is possible as direct predation on eggs and larvae, and by competition for zooplankton prey^1^. To calculate a calendar years’ worth of zooplankton data we took yearly averages of zooplankton starting in September (herring spawning time)^61^, discretized into low and high categories based on median values for each decade, and compared them to the zooplankton data the following year (Table 2). To rank the groups, the herring catch data were included in the zooplankton abundance network, linked via an edge to a zooplankton (and environmental) group, and the network score calculated. This scoring was repeated for all groups across the three decades. For each decade the rank of the biogeographical zooplankton group was given by the change in network score between the CPR networks without herring and the networks that included a forced link between the herring and the group. Note that these analyses make no assumptions or suggestions to whether the herring did depend on any zooplankton, instead they rank the relative importance of each group, so provide a different approach to that used with the jellyfish data.

## Results

### Irish Sea contemporary spatial network from cruise data

**Figure 5:**
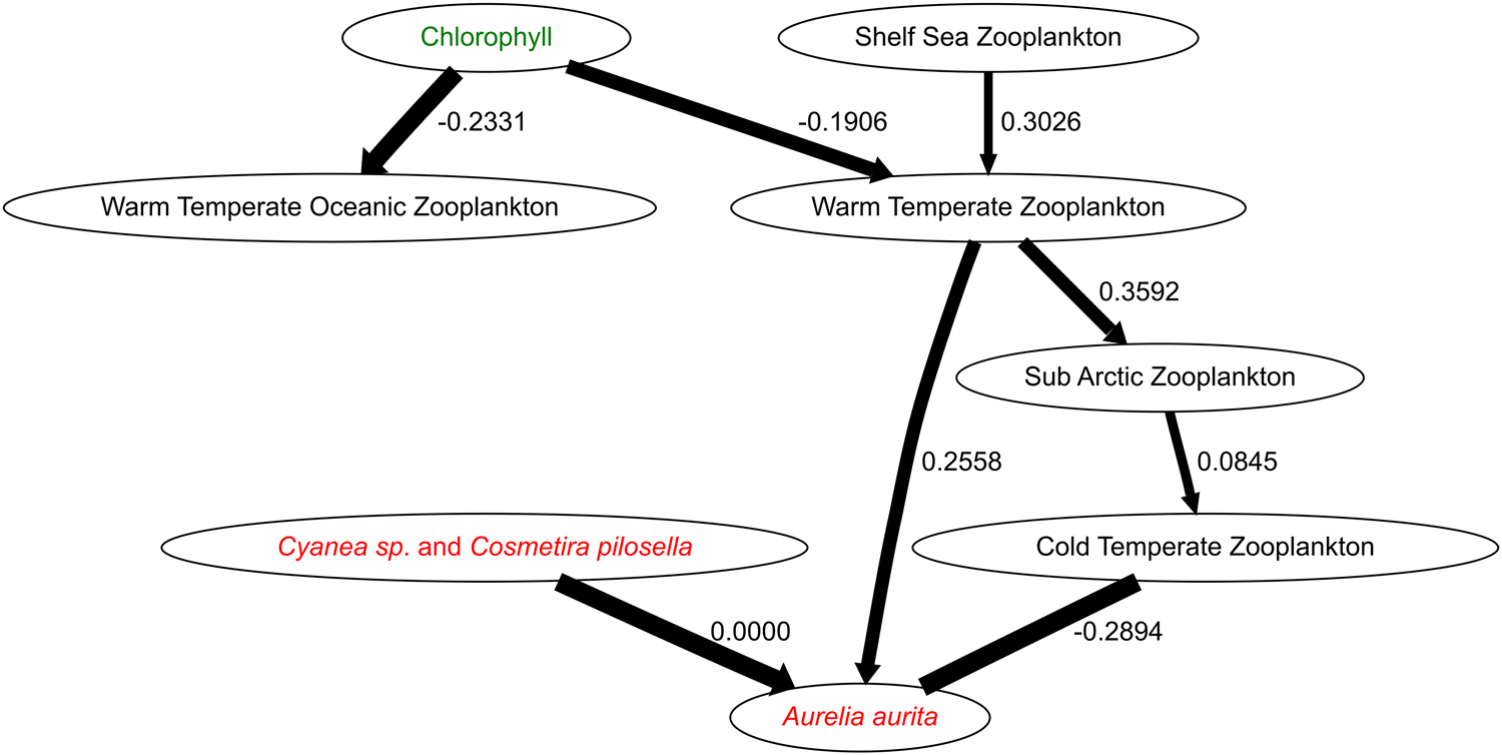
Jellyfish—zooplankton network apparent in the 2008 cruise data. The occurrence rate is indicated by the width of the edge (the line depicting dependencies between two taxa) - the wider the line, the higher the occurrence rate. Arrows indicate non-mutual dependence between two taxa; for example the Sub Arctic Zooplankton group has a positive dependency (is aggregated) with Warm Temperate Oceanic Zooplankton, but Warm Temperate Oceanic Zooplankton do not have a dependency with Sub Arctic Zooplankton. Where there is a mutual dependency between two groups, such as with Cold Temperate Oceanic Zooplankton and *Aurelia aurita,* the edge does not have an arrow. Numbers by the lines are the mean interaction strengths of the dependencies, with positive interaction strengths indicating aggregation, negative interaction strengths indicating segregation, and zero indicating different aggregation and segregation behaviours at different densities. Phytoplankton abundances are given by Chlorophyll Concentration.

The Bayesian Network for the field data collected in 2008 connected all nodes (Fig. 5). *Aurelia* was dependent on Cold Temperate zooplankton, Oceanic and Warm Temperate zooplankton, and on the *Cyanae* spp. and *Cosmetira pilosella* jellyfish group. Note that because these two zooplankton groups are connected to other zooplankton groups, the implication is that *Aurelia* will be effected indirectly by all these other zooplankton groups too^38^. The dependency of *Aurelia* with Warm Temperate Zooplankton was negative: high Warm Temperate Zooplankton corresponded to low *Aurelia* abundances whereas dependence on the Cold Temperate Oceanic Zooplankton group was non-monotonic, i.e. had different effects dependent on zooplankton abundances. Three of the edges between the zooplankton, Chlorophyll Concentration (indicating phytoplankton) and jellyfish groups showed positive dependences, three negative and two non-monotonic (Fig. 5).

### Historic zooplankton networks from CPR data

**Figure 6:**
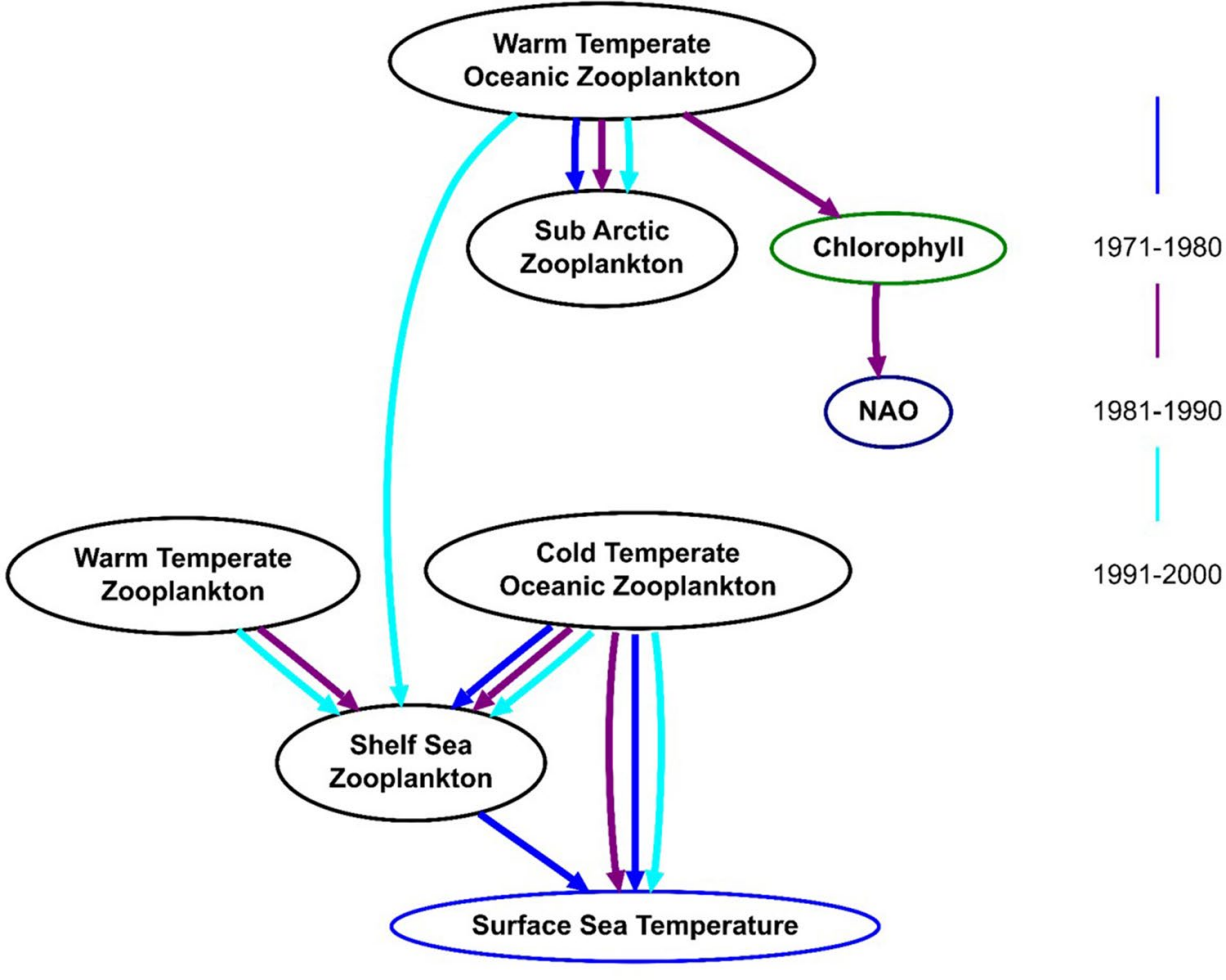
Historic zooplankton networks for the three time periods. Arrows indicate non-mutual dependence between two taxa; for example Shelf Sea Zooplankton are aggregated with respect to Warm Temperate Zooplankton, but Warm Temperate Zooplankton are not aggregated with respect to Shelf Sea Zooplankton.

The Bayesian Networks for CPR-sampled zooplankton across the three decades have different dependencies (Fig. 6). There are two sub-networks for the 1970s and 1980s networks, but these join into a single network for the 1990s. Three dependencies are present throughout the three networks: Shelf Sea Zooplankton with Cold Temperate zooplankton; Cold Temperate Zooplankton with SST, and Warm Temperate Oceanic zooplankton with Sub Arctic Zooplankton. The merge of the two sub-networks from the 1970s and 1980s in the 1990s occurs via the inclusion of the Warm Temperature Zooplankton in the 1980s network, and with the connection of Warm Temperate Oceanic Zooplankton to Shelf Sea zooplankton in the 1990s.

In order to investigate whether the changes in network structure were statistically significant, tests were conducted on the occurrence of edges for the three different time periods. There was no significant difference between the 1980s and 1990s (*p* = 0.3076), but the 1970s were significantly different to the 1980s (*p* = 0.0211) and 1990s (*p* = 0.0156). The network for 1981–1990 represents the time period over which zooplankton community composition has been recorded as changing elsewhere in the North Atlantic^49,53,62^. The 1981–1990 network is the only network where the Chlorophyll Concentration and NAOI variables are connected to other variables: this connection is via the Warm Temperate Oceanic Zooplankton group.

Changes to the ecological networks over time were also evident as changes in the strengths of the dependencies, as measured by the Interaction Strength (IS). The Shelf Sea Zooplankton and Cold Temperate zooplankton IS did not change significantly (as assessed by Chi-squared test) between 1970–1980 and 1981–1990, however it did change significantly (*p* < 0.0001) between the last two decades. Warm Temperature Oceanic Zooplankton and Sub Arctic Zooplankton changed significantly (*p* < 0.0001) between all three periods, with mean ISs of 0.44, 0.17 and 0.53 for the 1970s, 1980s and 1990s respectively. The IS for Cold Temperate Zooplankton and SST increased significantly over the three time periods, increasing sequentially from 0.24 to 0.47 and then to 0.64 (*p* < 0.0001).

### Ranking of relative importance of zooplankton groups to herring

The naïve Bayes classifiers found that the ranking of zooplankton groups with respect to influence on herring changed across the regime shift period (Table 3). The network improved pre-regime shift (1970s) with the additions of Cold Temperate Zooplankton to herring (ΔS = − 1.9110) and NAOI to herring (ΔS = − 0.5248), reflecting underlying relationships inferred for our analyses between the time-lagged herring catch data, zooplankton and the environment. Post regime shift (1990s), Cold Temperate Zooplankton were ranked fourth down from 3rd pre regime shift i.e. were 4th most influential on herring, but did not change the network score (ΔS = 0.0000), suggesting a loss of influence, while the NAOI remained ranked second and Warm Temperate Oceanic Zooplankton were ranked first (ΔS = − 4.0144). The NAOI influence score changed from positive pre regime-shift to negative post regime-shift. This change coincides with a shift in the NAO from a low phase to a high phase between the 1960s and 1990s^63^.

**Table 3:** The naïve Bayes classifiers for the time-lagged herring catch data with each group.

70s			80s			90s		
Group	Score change	IS with herring	Group	Score change	IS with herring	Group	Score change	IS with herring
Cold Temperate	− 1.9110	− 0.5455	Sub Arctic	− 0.9602	− 0.3077	Warm Temperate Ocean	− 4.0144	0.0000
NAO	− 0.7548	0.3791	Chlorophyll Concentration	-0.4704	− 0.3262	NAO	− 1.3775	− 0.4952
Sub Arctic	0.0000	− 0.1818	Cold Temperate	0.0000	0.125	Sub Arctic	− 0.9555	− 0.3636
Chlorophyll Concentration	0.0000	− 0.1818	Shelf Sea	0.0000	0.125	Cold Temperate	0.0000	0.0000
Warm Temperate Ocean	0.5628	− 0.1818	NAO	0.0000	− 0.5353	Chlorophyll Concentration	0.0000	− 0.3636
Temperature	0.7548	− 0.1818	Warm Temperate	0.4704	0.0000	Temperature	2.3330	0.0000
Warm Temperate	0.9055	− 0.6593	Warm Temperatecean	0.9602	0.125	Warm Temperate	2.7132	0.3808
Shelf Sea	1.9110	0.1818	Temperature	2.0665	− 0.286	Shelf Sea	3.0000	0.0000

The more negative the change in score, the better the network fit to the data, which reflects an underlying dependency between the herring and the group.

### Inference and probabilities

The Warm Temperate and Cold Temperate Oceanic Zooplankton groups have higher abundances at high SST (Table 4). The probability of being in a high abundance state given either high or low SST increases through time for the Warm Temperate Zooplankton group. In contrast, the abundance of the Cold Temperate Oceanic Zooplankton group remained steady throughout the three time periods given low SST, but decrease given high SST.

**Table 4:** Probability of *Aurelia* being in a low or high abundance state (excluding zero counts) given either low or high sea surface temperature over the three decades.

	70s		80s		90s	
	Low SST	High SST	Low SST	High SST	Low SST	High SST
Low *Aurelia*	13.42%	67.36%	18.32%	61.64%	17.73%	61.44%
High *Aurelia*	86.58%	32.64%	81.68%	38.36%	82.27%	38.56%

**Figure 7:**
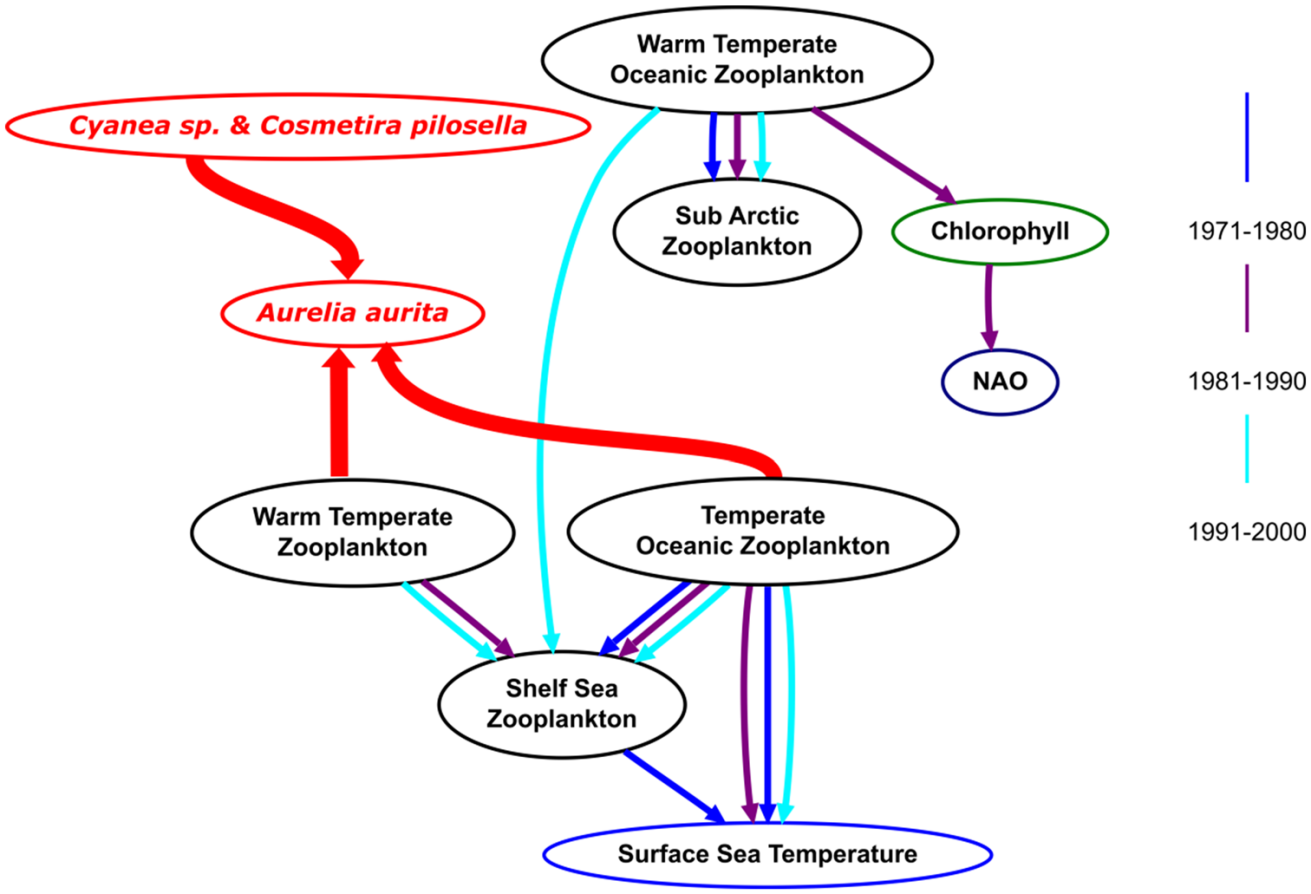
The network used to infer historic *Aurelia* abundances across the three decades (70s: purple edges, 80s: blue edges and 90s: cyan edges). The red edges and nodes were inferred from the cruise data, and are assumed to be constant over the historic study period (1971–2000). Different coloured edges represent different time periods.

Across the three decades, low SST corresponded to high inferred jellyfish probability, and high SST to low inferred jellyfish probability (Table 4). For both high and low inferred *Aurelia* abundance the probability of being in a high abundance state remained relatively constant, in contrast to the abundances of the zooplankton groups which changed significantly over the regime shift (Fig. 7).

## Discussion

Jellyfish-abundance time-series globally are rare, with less than 40 datasets over 10 years in length as of 2013^2^. Most of these datasets are from the northern hemisphere (87%), and in particular the Atlantic Ocean (17%) and the Mediterranean Sea (17%)^2^, so our understanding of how jellyfish abundances are changing globally through time is extremely limited. The debate on changing frequency of jellyfish bloom occurrences is however lively and ongoing, with a perception that the frequency of blooms is increasing e.g. Ref.^64^ due to anthropogenic influences such a overfishing and climate change^6,65^. While increases in jellyfish abundance have been found in some areas^2^, including in the Irish Sea^1,^ the misreporting of increased jellyfish blooms has led to over-stated generalisations on the occurrence of bloom events^66,67^. Global analyses of the available jellyfish time-series in 2013 showed a negligible overall increase with time, with a weak increasing linear trend that was possibly just an up-phase on a longer-period global oscillation^2^: more data and analyses are needed in order to tease apart the potentially complex interplay of jellyfish with other marine life and environmental variability.

In order to extend time-series of jellyfish abundance to a broader temporal and geographical scale, we propose an eco-palaeontological method for inferring historic jellyfish abundance from historic zooplankton data. We demonstrated this approach here by first inferring ecological zooplankton networks for three decades using historic CPR data from the Irish Sea from 1971 to 2000. Our BNI showed marked changes in zooplankton networks across these three decades, but three dependencies remained throughout: Cold Temperate Oceanic Zooplankton with SST and with Shelf Sea Zooplankton, and Warm Temperate Oceanic zooplankton and Sub Arctic Zooplankton (Fig. 6). In the 1980s, environmental factors became significantly more important—it was the only decade for which significant dependencies between a zooplankton group (Warm Temperate Oceanic Zooplankton) and Chlorophyll Concentration and an index of the NAO were found, possibly due to the regime shift perturbing ecosystem function. The regime shift resulted in fundamental changes to the historic zooplankton networks. Prior to 1990, the Warm Temperate Oceanic and Sub Arctic plankton did not depend on the other zooplankton groups, nor SST. As such, prior to 1990, there are two distinct ecological networks of zooplankton so that changes abundances within one network would not have affected the other network. Then, after the regime shift of the 1980s, in the 1990s Shelf Sea Zooplankton formed a dependency with Warm Temperate Oceanic Zooplankton, after the regime shift of the 1980s, so that changing abundances of any zooplankton group began to have knock-on effects across all the other groups. This increased connectivity likely increased ecosystem stability^68,69^ by potentially providing a broader spectrum of prey to predators of zooplankton.

We inferred the likely jellyfish—zooplankton networks for each of the three study decades (1970s, 80s and 90s) by adding the jellyfish—zooplankton interactions determined from the 2008 survey data networks to the historic CPR networks. Finally, we inferred *Aurelia* abundances for each of the three decades from the historic networks (Fig. 7). We found that inferred *Aurelia* abundances were not markedly impacted by changing zooplankton networks: T the probability of *Aurelia* existing in a high-abundance state being significantly higher for colder sea surface temperatures. This higher abundance state occurring despite significant changes over time in the abundance probabilities and Bayesian network structures for multiple zooplankton groups (Figs. 5, 7). It thus appears as though *Aurelia aurita* has been resilient to change in the composition of the zooplankton community, which is its prey. Our finding here that SST in the Irish Sea has a stronger influence on abundance of *A. aurita* than does the composition of the prey fields is consistent with a previous study that showed, using CPR data, that temperature was a more significant driver of the abundance of shelf jellyfish in the northern Benguela upwelling ecosystem than was food^16^.

From our results alone it is not possible to determine the underlying basis of the apparent resilience of *Aurelia aurita* to the mid 1980's regime shift. One possibility is whilst the species composition of the Irish Sea zooplankton community changed, the size spectrum of that community and the nutritional content did not change, so *Aurelia*′s preyfield remained functionally similar. Although the regime shift in the 1980s saw changes in species composition and ecological network structure, ecosystem stability and resilience from the perspective of *Aurelia* was maintained via various processes including functional and/or trophic redundancy^70,71^, competition trade-offs^72^, stabilizing feedback loops^36^ and/or functional complexity^73^. Despite this putative functional similarity in the zooplankton cimmunity from the perspective of *Aurelia*, there were though major differnces in zooplankton network connectivity pre- and post regime shift. The post regime-shift network (1990s) was the only one of the three decades where all zooplankton groups connected (Fig. 7). The increased connectivity within the 1990s zooplankton network may be due to the decreasing month-specificity of zooplankton group peaks, with a broadening of the abundance peaks for most zooplankton groups, resulting in an increased temporal overlap (Fig. 4). In the context of Cushing’s match-mismatch hypothesis, this may give *Aurelia* a longer time window each year to surf the wave of zooplankton production. We suggest that the increased connectivity between different biogeographical zooplankton groups created redundancy in prey species, and so enabled prey-switching by *Aurelia*^74^. *Aurelia* had dependencies with the Warm Temperate and Cold Temperate Oceanic Zooplankton groups, in which cyphyonautes larvae dominate the Warm Temperate group and *Calanus helgolandicus* and barnacle larvae dominate the Cold Temperate Oceanic zooplankton group. Therefore, it is likely that these taxa are the key drivers for this network, with *Aurelia* switching prey post regime shift. In our data, particularly abundant members of the Warm Temperate Zooplankton group included cyphonaute larvae of bryozoans and numerous species of mesozooplanktic copepods. *Aurelia* spp. are known to be able to thrive on very small zooplankton^75^ and indeed feeding experiments on *Aurelia aurita* from the Black Sea suggest that food items overlooked by researchers appraising prey visually may provide ten times the energy for *Aurelia* than that obtained from mesozooplankton^76^: it is possible that *Aurelia* medusa could be sustained by cyphonautes. Another possibility is that environmental conditions that favour strobilation (asexual reproduction) of *Aurelia* benthic phases also favour bryozoan reproduction. Our own work, for example, has shown that cold winter conditions lead to elevated rates of ephyrae production by strobilation^77^ and that abundance of medusae in the North Sea is high in years characterised with low NAO index. A similar association is evident between NAO phase and abundance of cyphonaute larvae in Loch Hyne^78^.

There was no clear association between *Aurelia* abundance probabilities (Table 4) and herring catch (Fig. 1), which suggests that *Aurelia* abundances and herring recruitment do not directly interact. This lack of interaction can be explained by our 2008 field-data network, which did not include any direct dependencies between *Aurelia* (or any other jellyfish) and the Sub Arctic Zooplankton group (Fig. 6) which is dominated by *Calanus finmarchicus* (Table 1), historically the primary food source of herring^79^. The ranked zooplankton groups (Table 3) show that the Cold Temperate Zooplankton (*Acartia* spp., *Podon* spp. and *Evadne* spp.) was the most important group prior to the regime shift, and afterwards changed to Cold Temperate Oceanic (dominated by cyphyonaute larvae) and Sub-Artic Zooplankton group (dominated by *Calanus finmarchicus*). It appears that *Aurelia* do not compete directly with herring for larger stages of *Calanus finmarchicus,* and while small herring may compete to some extent with *Aurelia* for the *cyphyonaute* larvae, these larvae historically formed only a relatively small proportion (5.4%)^80^ of the herring diet so are unlikely to have a strong influence on herring success.

Our conclusions on jellyfish—herring interactions in the Irish Sea differ from those drawn by previous studies of the North Sea, which found that over the period 1958–2007 jellyfish were negatively impacting herring recruitment^4,7^. The biogeographical distribution of *Calanus finmarchicus* changed significantly in the North Sea during the mid-1980s^24^, suggesting that Irish Sea herring would have experienced a less substantial change in their prey field during this time. In any case our networks suggest minimal interaction between *Calanus finmarchicus* in the Irish Sea. It would be instructive to run a network analysis of North Sea zooplankton and jellyfish, but that is beyond the scoipe of the present study (Fig. 6).

Plankton communities in the Irish Sea, like as in the North Sea, are primarily driven by hydroclimatic conditions^24^, with some areas now showing increased autumnal diatom production due to anthropogenic nutrient enrichment^81^. Future anthropogenic impact and climatic changes are likely to lead to further changes for Irish Sea plankton communities. These changes are likely to include further zooplankton biogeographic shifts, with cold-water species being replaced by the warm-water species as  the cold-water species retreat north. It is not possible to predict from our analyses here how these changes will affect jellyfish abundances, and as jellyfish responses would depend on whether further changes to network structure occur. However, jellyfish abundances may not necessarily increase: from our inferred historic jellyfish abundances there are noticeably higher abundances with cold sea surface temperature, so any increased warming may have a negative impact on jellyfish abundances, despite their resilience over the 30 year study period here^5^.

Our BNI technique has provided a window into the past and revealed a new perspective on jellyfish ecosystem interactions in the Irish Sea. Using a combination of historic zooplankton data with contemporary jellyfish data we have been able to demonstrate that, despite a change in the ecological networks of zooplankton between the 1970s and 1990s, the abundance of *Aurelia aurita* most likely remained stable: we suggest that is a consequence of *Aurelia*′s generalist ability to take a wide range of prey. Our work suggests a remarkable resilience of *Aurelia aurita* to ecosystem reorganisation experienced in the Irish Sea over the regime shift of the 1980s. The generalist ability of *Aurelia* as exhibited in the Irish Sea may help explain the pan-global distribution of the Moon jellyfish species complex and its success in a diversity of locations^82,83^.

## Data Availability

Data are available on Figshare: 10.6084/m9.figshare.12620753.

## Code availability

Code is available on https://github.com/egmitchell/bootstrap , https://github.com/egmitchell/jellyfish.

## References

[1] Lynam CP (2011). Have jellyfish in the Irish Sea benefited from climate change and overfishing?. Glob. Change Biol..

[2] Condon RH (2013). Recurrent jellyfish blooms are a consequence of global oscillations. Proc. Natl. Acad. Sci..

[3] Lynam CP, Hay SJ, Brierley AS (2005). Jellyfish abundance and climatic variation: Contrasting responses in oceanographically distinct regions of the North Sea, and possible implications for fisheries. J. Mar. Biol. Assoc..

[4] Lynam CP (2006). Jellyfish overtake fish in a heavily fished ecosystem. Curr. Biol..

[5] Hays GC, Doyle TK, Houghton JDR (2018). A paradigm shift in the trophic importance of jellyfish?. Trends Ecol. Evol..

[6] Gibbons, M. J. & Richardson, A. J. Patterns of jellyfish abundance in the North Atlantic. In *Jellyfish Blooms: Causes, Consequences, and Recent Advances: Proceedings of the Second International Jellyfish Blooms Symposium, held at the Gold Coast, Queensland, Australia, 24–27 June, 2007* (eds. Pitt, K. A. & Purcell, J. E.) 51–65 (Springer Netherlands, 2009). 10.1007/978-1-4020-9749-2_4.

[7] Attrill MJ, Wright J, Edwards M (2007). Climate-related increases in jellyfish frequency suggest a more gelatinous future for the North Sea. Limnol. Oceanogr..

[8] Brodeur RD, Sugisaki H, Hunt GL (2002). Increases in jellyfish biomass in the Bering Sea: Implications for the ecosystem. Mar. Ecol. Prog. Ser..

[9] Pauly D, Christensen V, Dalsgaard J, Froese R, Torres F (1998). Fishing down marine food webs. Science.

[10] Purcell JE, Arai MN (2001). Interactions of pelagic cnidarians and ctenophores with fish: A review. Hydrobiologia.

[11] Robinson KL (2014). Jellyfish, forage fish, and the world’s major fisheries. Oceanography.

[12] Uye S (2008). Blooms of the giant jellyfish *Nemopilema nomurai*: A threat to the fisheries sustainability of the East Asian Marginal Seas. Plankton Benthos Res..

[13] Wright RM, Le Quéré C, Buitenhuis E, Pitois S, Gibbons M (2020). Unique role of jellyfish in the plankton ecosystem revealed using a global ocean biogeochemical model. Biogeosci. Discuss..

[14] Jackson JBC (2008). Ecological extinction and evolution in the brave new ocean. Proc. Natl. Acad. Sci..

[15] Kintner A, Brierley AS (2019). Cryptic hydrozoan blooms pose risks to gill health in farmed North Atlantic salmon (*Salmo salar*). J. Mar. Biol. Assoc..

[16] Flynn BA (2012). Temporal and spatial patterns in the abundance of jellyfish in the northern Benguela upwelling ecosystem and their link to thwarted pelagic fishery recovery. Afr. J. Mar. Sci..

[17] Luo JY (2020). Gelatinous zooplankton-mediated carbon flows in the global oceans: A data-driven modeling study. Glob. Biogeochem. Cycles.

[18] Behrenfeld MJ (2006). Climate-driven trends in contemporary ocean productivity. Nature.

[19] Hays GC, Richardson AJ, Robinson C (2005). Climate change and marine plankton. Trends Ecol. Evol..

[20] Richardson AJ, Schoeman DS (2004). Climate impact on plankton ecosystems in the northeast Atlantic. Science.

[21] Suikkanen S (2013). Climate change and eutrophication induced shifts in northern summer plankton communities. PLoS ONE.

[22] Wiafe G, Yaqub HB, Mensah MA, Frid CLJ (2008). Impact of climate change on long-term zooplankton biomass in the upwelling region of the Gulf of Guinea. ICES J. Mar. Sci..

[23] Reid PC, Colebrook JM, Matthews JBL, Aiken J (2003). The Continuous Plankton Recorder: Concepts and history, from Plankton Indicator to undulating recorders. Prog. Oceanogr..

[24] Edwards M (2020). Plankton, jellyfish and climate in the North-East Atlantic. MCCIP Sci. Rev..

[25] ICES. *Report of the Working Group on the Celtic Seas Ecoregion (WGCSE), 11–19 May 2011, Copenhagen, Denmark*. (2011).

[26] Bartolino, V. *et al.* Herring assessment working group for the area south of 62° N (HAWG). (2019) 10.17895/ices.pub.5460.

[27] *ICES Advice Book 5*. https://www.ices.dk/sites/pub/Publication%20Reports/Advice/2007/may/her-nirs.pdf (2007).

[28] Beaugrand G (2004). The North Sea regime shift: Evidence, causes, mechanisms and consequences. Prog. Oceanogr..

[29] Gregory B, Christophe L, Martin E (2009). Rapid biogeographical plankton shifts in the North Atlantic Ocean. Glob. Change Biol..

[30] deYoung B (2008). Regime shifts in marine ecosystems: Detection, prediction and management. Trends Ecol. Evol..

[31] Bastian T (2011). Large-scale sampling reveals the spatio-temporal distributions of the jellyfish *Aurelia aurita* and *Cyanea capillata* in the Irish Sea. Mar. Biol..

[32] Houghton JDR, Doyle TK, Davenport J, Hays GC (2006). Developing a simple, rapid method for identifying and monitoring jellyfish aggregations from the air. Mar. Ecol. Prog. Ser..

[33] Bastian T, Lilley MKS, Beggs SE, Hays GC, Doyle TK (2014). Ecosystem relevance of variable jellyfish biomass in the Irish Sea between years, regions and water types. Estuar. Coast. Shelf Sci..

[34] Heckerman D, Geiger D, Chickering DM (1995). Learning Bayesian networks: The combination of knowledge and statistical data. Mach. Learn..

[35] Milns I, Beale CM, Smith VA (2010). Revealing ecological networks using Bayesian network inference algorithms. Ecology.

[36] Mitchell EG, Neutel A-M (2012). Feedback spectra of soil food webs across a complexity gradient, and the importance of three-species loops to stability. Theor. Ecol..

[37] Olff H (2009). Parallel ecological networks in ecosystems. Philos. Trans. R. Soc. B Biol. Sci..

[38] Mitchell EG, Whittle R, Griffths HJ (2020). Benthic ecosystem cascade effects in Antarctica using Bayesian network inference. Commun. Biol..

[39] Yu J, Smith VA, Wang PP, Hartemink AJ, Jarvis ED (2004). Advances to Bayesian network inference for generating causal networks from observational biological data. Bioinformatics.

[40] Yu, J., Smith, V. A., Wang, P. P., Hartemink, E. J. & Jarvis, E. D. Using Bayesian network inference algorithms to recover molecular genetic regulatory networks. In *Prof. of Int*. (2002).

[41] Smith VA, Yu J, Smulders TV, Hartemink AJ, Jarvis ED (2006). Computational inference of neural information flow networks. PLOS Comput. Biol..

[42] Mitchell EG, Butterfield NJ (2018). Spatial analyses of Ediacaran communities at Mistaken Point. Paleobiology.

[43] Mitchell EG, Durden JM, Ruhl HA (2020). First network analysis of interspecific associations of abyssal benthic megafauna reveals potential vulnerability of abyssal hill community. Prog. Oceanogr..

[44] Mitchell EG, Harris S (2020). Mortality, population and community dynamics of the glass sponge dominated community “The Forest of the Weird” from the RIDGE seamount, Johnston Atoll, Pacific Ocean. Front. Mar. Sci..

[45] Reid PC, Borges MDF, Svendsen E (2001). A regime shift in the North Sea circa 1988 linked to changes in the North Sea horse mackerel fishery. Fish. Res..

[46] Brierley AS (2001). Acoustic observations of jellyfish in the Namibian Benguela. Mar. Ecol. Prog. Ser..

[47] Brierley AS (2005). Towards the acoustic estimation of jellyfish abundance. Mar. Ecol. Prog. Ser..

[48] MacLennan DN, Simmonds EJ (2013). Fisheries Acoustics.

[49] Planque B, Fromentin J (1996). Calanus and environment in the eastern North Atlantic. I. Spatial and temporal patterns of *C. finmarchicus* and *C. helgolandicus*. Mar. Ecol. Prog. Ser..

[50] Batten SD (2003). CPR sampling: The technical background, materials and methods, consistency and comparability. Prog. Oceanogr..

[51] Richardson AJ (2006). Using continuous plankton recorder data. Prog. Oceanogr..

[52] John EH (2002). Continuous plankton records stand the test of time: Evaluation of flow rates, clogging and the continuity of the CPR time-series. J. Plankton Res..

[53] Beaugrand G, Ibañez F, Lindley JA, Reid PC (2002). Diversity of calanoid copepods in the North Atlantic and adjacent seas: Species associations and biogeography. Mar. Ecol. Prog. Ser..

[54] Yu J (2005). Developing Bayesian Network Inference Algorithms to Predict Causal Functional Pathways in Biological Systems.

[55] Chickering, D. M. Learning Bayesian Networks is NP-Complete. In *Learning from Data: Artificial Intelligence and Statistics V* (eds. Fisher, D. & Lenz, H.-J.) 121–130 (Springer, Berlin, 1996). 10.1007/978-1-4612-2404-4_12.

[56] Rayner, N. A. *et al.* Global analyses of sea surface temperature, sea ice, and night marine air temperature since the late nineteenth century. *J. Geophys. Res. Atmos.* **108**, (2003).

[57] Jones PD, Jonsson T, Wheeler D (1997). Extension to the North Atlantic oscillation using early instrumental pressure observations from Gibraltar and south-west Iceland. Int. J. Climatol..

[58] Mitchell EG (2011). Functional programming through deep time: Modeling the first complex ecosystems on earth. ACM SIGPLAN Not..

[59] Jones SP (2003). Haskell 98 Language and Libraries: The Revised Report.

[60] Friedman N, Geiger D, Goldszmidt M (1997). Bayesian network classifiers. Mach. Learn..

[61] Dickey-Collas M, Nash RDM, Brown J (2001). The location of spawning of Irish sea herring (*Clupea harengus*). J. Mar. Biol. Assoc..

[62] Nash RDM, Geffen AJ (2004). Seasonal and interannual variation in abundance of *Calanus finmarchicus* (Gunnerus) and *Calanus helgolandicus* (Claus) in inshore waters (west coast of the Isle of Man) in the central Irish Sea. J. Plankton Res..

[63] Hurrell JW, Deser C (2009). North Atlantic climate variability: The role of the North Atlantic Oscillation. J. Mar. Syst..

[64] Brotz, L., Cheung, W. W. L., Kleisner, K., Pakhomov, E. & Pauly, D. Increasing jellyfish populations: trends in Large Marine Ecosystems. In *Jellyfish Blooms IV: Interactions with Humans and Fisheries* (eds. Purcell, J. *et al.*) 3–20 (Springer Netherlands, 2012). 10.1007/978-94-007-5316-7_2.

[65] Purcell JE (2012). Jellyfish and ctenophore blooms coincide with human proliferations and environmental perturbations. Annu. Rev. Mar. Sci..

[66] Pitt KA, Lucas CH, Condon RH, Duarte CM, Stewart-Koster B (2018). Claims that anthropogenic stressors facilitate jellyfish blooms have been amplified beyond the available evidence: A systematic review. Front. Mar. Sci..

[67] Sanz-Martín M (2016). Flawed citation practices facilitate the unsubstantiated perception of a global trend toward increased jellyfish blooms. Glob. Ecol. Biogeogr..

[68] Dunne JA, Williams RJ, Martinez ND (2002). Network structure and biodiversity loss in food webs: Robustness increases with connectance. Ecol. Lett..

[69] Gardner MR, Ashby WR (1970). Connectance of large dynamic (cybernetic) systems: Critical values for stability. Nature.

[70] Lawton, J. H. & Brown, V. K. Redundancy in ecosystems. In *Biodiversity and Ecosystem Function* (eds. Schulze, E.-D. & Mooney, H. A.) 255–270 (Springer, Berlin, 1994). 10.1007/978-3-642-58001-7_12.

[71] Thébault E, Loreau M (2005). Trophic interactions and the relationship between species diversity and ecosystem stability. Am. Nat..

[72] Wang S, Loreau M (2016). Biodiversity and ecosystem stability across scales in metacommunities. Ecol. Lett..

[73] Van Voris P, O’Neill RV, Emanuel WR, Shugart HH (1980). Functional complexity and ecosystem stability. Ecology.

[74] Graham WM, Kroutil RM (2001). Size-based prey selectivity and dietary shifts in the jellyfish, *Aurelia aurita*. J. Plankton Res..

[75] Marques, R., Bonnet, D., Carré, C., Roques, C. & Darnaude, A. M. Trophic ecology of a blooming jellyfish (*Aurelia coerulea*) in a Mediterranean coastal lagoon. *Limnol. Oceanogr.* **n/a**.

[76] Anninsky, B. E., Finenko, G. A., Datsyk, N. A. & Kıdeyş, A. E. Trophic ecology and assessment of the predatory impact of the Moon jellyfish Aurelia aurita (Linnaeus, 1758) on zooplankton in the Black Sea (2020) 10.21411/cbm.a.96dd01aa.

[77] Widmer CL, Fox CJ, Brierley AS (2016). Effects of temperature and salinity on four species of northeastern Atlantic scyphistomae (Cnidaria: Scyphozoa). Mar. Ecol. Prog. Ser..

[78] Watson DI, Barnes DKA (2004). Temporal and spatial components of variability in benthic recruitment, a 5-year temperate example. Mar. Biol..

[79] Arrhenius F, Hansson S (1993). Food consumption of larval, young and adult herring and sprat in the Baltic Sea. Mar. Ecol. Prog. Ser..

[80] Williams R, Conway DVP, Hunt HG (1994). The role of copepods in the planktonic ecosystems of mixed and stratified waters of the European shelf seas. Hydrobiologia.

[81] Gowen RJ, Mills DK, Trimmer M, Nedwell DB (2000). Production and its fate in two coastal regions of the Irish Sea: The influence of anthropogenic nutrients. Mar. Ecol. Prog. Ser..

[82] Scorrano S, Aglieri G, Boero F, Dawson MN, Piraino S (2017). Unmasking Aurelia species in the Mediterranean Sea: An integrative morphometric and molecular approach. Zool. J. Linn. Soc..

[83] Haussermann V, Dawson MN, Forsterra G (2009). First record of the moon jellyfish, Aurelia for Chile. Spixana.

